# TLR2 Arg753Gln Gene Polymorphism Associated with Tuberculosis Susceptibility: An Updated Meta-Analysis

**DOI:** 10.1155/2019/2628101

**Published:** 2019-01-13

**Authors:** Lelin Hu, Huihui Tao, Xinrong Tao, Xiaolong Tang, Congjing Xu

**Affiliations:** ^1^Department of Clinical Medicine, School of Medicine, Anhui University of Science and Technology, Huainan 232001, Anhui, China; ^2^Department of Radiation Oncology, Huainan First People's Hospital, Huainan 232001, Anhui, China; ^3^Department of Cell Biology, School of Medicine, Anhui University of Science and Technology, Huainan 232001, Anhui, China; ^4^Department of Respiration, Tumor Hospital of Affiliated Huainan East Hospital Group, China

## Abstract

**Objective:**

To date, a series of studies were conducted to investigate the association between TLR2 (Toll-like receptor 2) Arg753Gln gene polymorphism and tuberculosis (TB). However, the results were inconsistent. This meta-analysis was performed to elucidate the roles of TLR2 Arg753Gln gene polymorphism in TB.

**Methods:**

All available articles were searched from online databases such as PubMed, Medline, CNKI, and Wanfang. Statistical analyses were performed using the STATA12.0 (Stata Corp LP, College Station, TX, United States) software.

**Results:**

32 case-control studies comprising 5943 cases and 5991 controls were identified in this meta-analysis. Overall, the TLR2 Arg753Gln gene polymorphism was associated with high TB risk in allele model (A vs. G: OR=2.20, 95%CI=1.60-3.04, P≤0.01), dominant model (AA+AG vs. GG: OR=2.70, 95%CI=2.00-3.65, P≤0.01), and heterozygote model (AG vs. GG contrast: OR=2.97, 95%CI=2.39-3.69, P≤0.01). Subgroup analysis by ethnicity indicated that the A allele increased susceptibility to TB in Asian (OR=3.35, 95%CI=2.36-4.74) and Caucasian populations (OR=2.62, 95%CI=1.77-3.87), but not in African (2.08, 95%CI=0.62-2.72) or mixed populations (OR=0.76, 95%CI=0.36-1.14). Stratified analysis by sample type suggested that the A allele associated with high pulmonary tuberculosis (PTB) risks (OR=2.43, 95%CI=1.66-3.54), but not with extra pulmonary tuberculosis (EPTB) (OR=1.84, 95%CI=0.83-4.06).

**Conclusion:**

this meta-analysis suggested the following: (1) TLR2 Arg753Gln polymorphism is significantly associated with high TB risk. (2) In subgroup analysis based on ethnicity, TLR2 Arg753Gln polymorphism elevates the risk of TB in Asian and Caucasian populations, but not in African or mixed populations. (3) Stratified by sample type, TLR2 Arg753Gln polymorphism is associated with increased PTB risk, but not with EPTB.

## 1. Introduction

Tuberculosis (TB) is an infectious disease caused by* Mycobacterium tuberculosis* (*M. tuberculosis*), which is the leading cause of death attributable to a single infectious agent worldwide [1]. The host innate immune response is important for the activation of the adaptive immune response and initial defense against* M. tuberculosis* [2]. Toll-like receptors (TLRs) are a family of proteins that are expressed either on the extra cellular cell surface (TLR1, 2, 4, 5, 6) or in the cytosol or on endosomal membranes (TLR3, 7, 8, 9) of macrophages and dendritic cells. TLRs play a key role in the innate immune response to infectious agents through discriminating self-pathogen-associated molecular patterns (PAMPs) such as lipopolysaccharide (LPS), teichoic acid, and surface lipoproteins and initiating NF-*κ*B signal transduction pathway [3]. Nuclear translocation of NF-*κ*B induces transcription of proinflammatory cytokine genes essential to mounting a protective immune response. Different TLRs can recognize distinct classes of products synthesized by pathogens and regulate host's defense against invading pathogens [4].

TLR2 is one of the most studied TLRs with regard to TB disease. It can recognize a wide range of mycobacterial PAMPs including triacylated lipopeptides and diacylated lipopeptides. It has been verified that defects in TLR2 could influence the formation of heterodimers with TLR1 and TLR6 and impede the recognition of corresponding receptors, thus increasing TB susceptibility. TLR2 encoding gene located on the long arm of chromosome 4(4q32), where several single-nucleotide polymorphisms (SNPs) have been identified. TLR2 Arg753Gln (rs5743708, G2258A) is one of the most characterized SNPs reported in previous functional studies. Recently, the effect of TLR2 Arg753Gln gene polymorphism on the development of TB has received increased attention. The variant of TLR2 Arg753Gln leads to a substitution of arginine to glutamine at residue 753, which results in a decreased response of macrophages to bacterial peptides. And the host susceptibility to TB sharply increases [5]. Over the last three decades, a series of case-control studies focus on the association between TLR2 Arg753Gln polymorphism and TB risk in human [2, 6–27]. However, the results of these studies are inconsistent. We performed this meta-analysis to assess the association between the most commonly investigated TLR2 Arg753Gln polymorphism and TB risk both across and within different ethnicities.

## 2. Materials and Methods

### 2.1. Inclusion and Exclusion Criteria

We searched all eligible articles in Medline, PubMed, Wangfang, and CNKI (China National Knowledge Infrastructure) databases to identify studies on the association between TLR2 Arg753Gln polymorphism and TB. All articles were published until July 1st, 2018. The key words were used for the following Medical Subject Heading (MeSH) terms: (“TLR2” or “Toll like receptor 2”) and (“polymorphism” or “allele” or “mutation” or “variant”) and (“tuberculosis” or “TB”). The search was conducted on human subject by two investigators independently. The reference of retrieved articles and reviews was tracked to identify other eligible studies. The study that lacked detailed allele or genotype information was excluded from the meta-analysis.

Ethical approval of the study was not required.

### 2.2. Data Extraction

**Figure 1:**
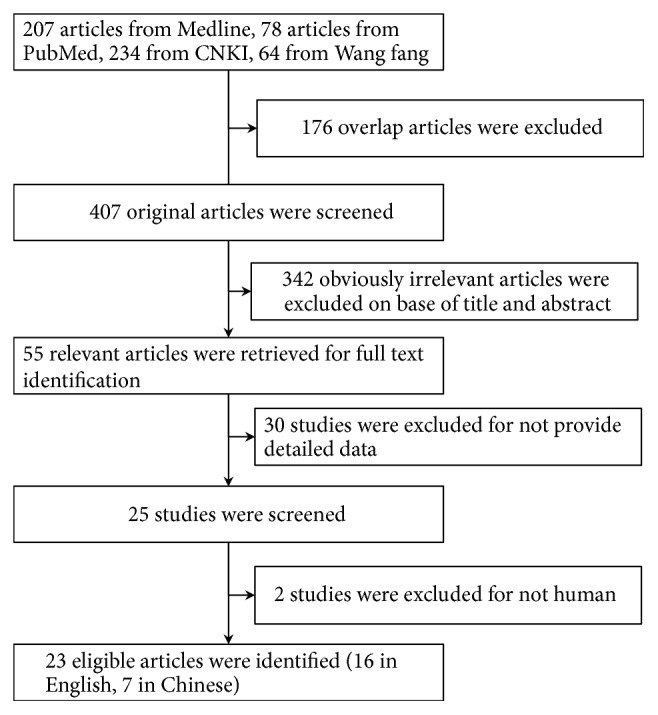
Flow diagram of selection process of eligible studies.

As shown in Figure 1, eligible studies were selected according to the following inclusion criteria: (1) case-control study, (2) study on the association between TLR2 polymorphism and TB, (3) detailed genotype distributions for both cases and controls being available. Studies were excluded if one of the following exclusion criteria existed: (1) the design was not on human subject; (2) the genotype distribution of TLR2 Arg753Gln was not reported; (3) the information for extraction of data was insufficient; (4) data was overlapping. The key information of each eligible study was recorded in detail as follows: author's name, the year of publication, the country of the study population, ethnicity, genotyping methods, sample type, genotype and allele distributions, and the number of cases and controls (Table 1).

### 2.3. Statistical Analysis

Available raw data were extracted from included studies. Deviation from Hardy-Weinberg equilibrium (HWE) in the controls was tested by Pearson's *χ*2 test (P<0.05 means deviated from HWE). ORs (the odds ratios), their 95% CIs (95% confidence intervals), and P value were calculated to evaluate the strength of association between TLR2 Arg753Gln polymorphism and the risk of TB in corresponding comparison models. The significance of pooled OR was determined by Z-test and P-value<0.05 was considered statistically significant.

*χ* ^2^-based Q statistics were used to test heterogeneity between studies. The heterogeneity among the studies was measured by I^2^ value and P value. If P<0.10, the random-effects model was chosen to calculate heterogeneity. Otherwise, the fixed-effect was used. Meta-analyses were performed in overall and in subgroup for ethnic groups or HWE. For sensitivity analysis, the omission of one study a time to assess relative influence of each study on the pooled. Begg's funnel plots and Egger's test were used to evaluate potential publication bias.

## 3. Results

### 3.1. Characteristics of Included Studies

32 eligible case-control studies from 23 original articles including a total of 5943 TB cases and 5991 controls for TLR2 Arg753Gln gene polymorphism were identified in this meta-analysis. The detailed characteristics of the included studies were shown in Table 1. The allele and genotype distributions of the TLR2 Arg753Gln polymorphism among TB were enlisted in Table 2. Of the 32 studies, 10 studies were conducted among Caucasian populations, seventeen among Asian populations, two among African populations, and three among mixed populations. Except for the study of Salie et al., the genotyping method of another 31 studies is available. 15 of these studies used classic PCR-RFLP (polymerase chain reaction-restriction fragment length polymorphism) assay, 5 studies used ARMS-PCR (the amplification refractory mutation system), 5 studies used DNA sequencing technique, and the 6 other studies used PCR-SSP (sequence specific primer), MALDI-TOFMS (matrix-assisted laser desorption/ionization time-of-flight mass spectrometry), SNaPshot, and Taqman. The genotype distributions in the controls of 19 included studies were in agreement with HWE, and the other 13 included studies were deviated from HWE. According to the sample type, there were 7 TB studies, 18 PTB studies, and 4 EPTB studies.

### 3.2. Meta-Analysis Results

The association between TLR2 Arg753Gln polymorphism and TB was examined in allele model (A vs. G), the dominant model (AA + GA vs. GG allele), recessive model (AA vs. GA + GG), homozygote model (AA vs.  GG), and heterozygote model (GA vs. GG). If the heterogeneity was obvious (P< 0.10), the random effects model was used, or else the fixed-effect model was applied. However, many studies were excluded in recessive model (AA vs. GA + GG) and homozygote model (AA vs.  GG) because the value of AA genotype is zero in these included studies. There are only 4 studies left in homozygote model (AA vs.  GG) and 4 studies left in recessive model (AA vs. GA + GG). Therefore, allele model (A vs. G), heterozygote model (GA vs. GG), and dominant model (AA + GA vs. GG) were used to assess the association between TLR2 Arg753Gln polymorphism and TB risk in this study.

**Figure 2:**
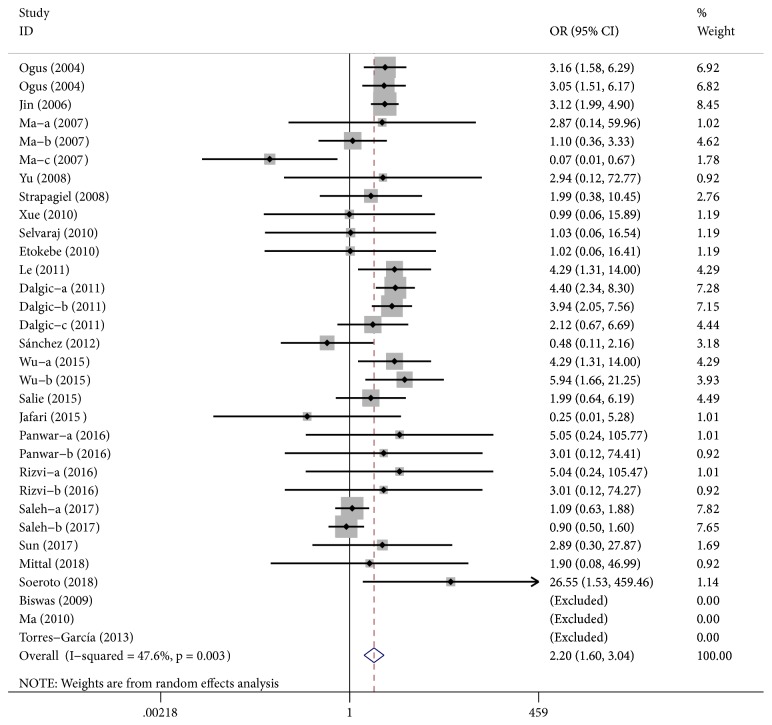
Forest plot of tuberculosis risk associated with TLR2 Arg753Gln polymorphism in allele genetic model.

A significantly increased TB risk was found in the overall population in allele genetic model (A vs. G: OR=2.39, 95%CI=2.02-2.84, P≤0.01, Figure 2), dominant genetic model (AA+AG vs. GG: OR=3.10, 95%CI=2.54-3.78, P≤0.01), and heterozygote genetic model (AG vs. GG: OR=3.08, 95%CI=2.52-3.77, P≤0.01) (Table 3). When stratified by ethnicity, a significant increased tuberculosis risk associated with TLR2 Arg753Gln polymorphism was found in allele genetic model among Caucasian or Asian populations. However, there was no association between TLR2 Arg753Gln polymorphism and TB among African populations (A vs. G: OR=2.08, 95%CI=0.72-2.02, P=0.18) or mixed populations (A vs. G: OR=0.76, 95%CI=0.36-1.64, P=0.49). In the subgroup analysis by sample type, TLR2 Arg753Gln polymorphism is associated with high pulmonary tuberculosis (PTB) risks, but not with EPTB or TB. For example, in allele genetic model, OR=2.43, 95%CI=1.66-3.54, P≤0.01 for PTB; OR=1.84, 95% CI= 0.83-4.06, P=0.13 for EPTB; OR=1.84, 95% CI=0.74-4.59, P=0.19 for TB. If the studies deviated from HWE were excluded, the results were not affected (Table 4).

### 3.3. Heterogeneity and Sensitivity Analysis

**Figure 3:**
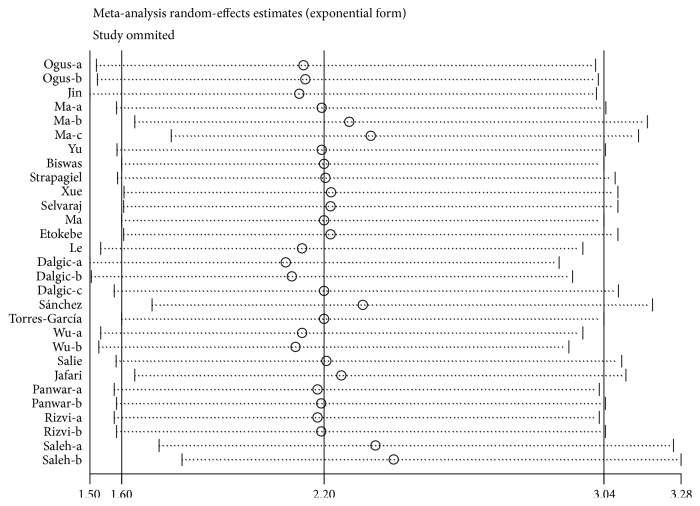
Sensitivity analyses for the influence of individual study on the pooled ORs.

As shown in Table 3, the heterogeneity was significant between studies in allele model for TLR2 Arg753Gln polymorphism (A vs. G: P(Q-test) P≤0.10), but not in dominant genetic model (AA+AG vs. GG: P(Q-test)=0.10) and heterozygote genetic model (AG vs. GG: P(Q-test)=0.18). The stratified analysis by HWE of controls, ethnicity, sample type in allele genetic model was performed to clarify the source of heterogeneity (Table 4). The results suggested that ethnicities might contribute to the heterogeneity. Stratified by ethnicity the heterogeneity was not statistically significant in Caucasian, Asian, and African populations, while being statistically significant in mixed populations. After excluding studies of mixed populations, the heterogeneity in overall was resolved (Figure S1). The sensitivity analysis was performed to assess the source of heterogeneity. The influence of a single study on the pooled ORs was investigated by omission of one study a time; the omission of any study made no significant qualitatively influence to the pooled ORs, indicating that the results were statistically reliable (for example, in allele genetic model, Figure 3).

### 3.4. Publication Bias

**Figure 4:**
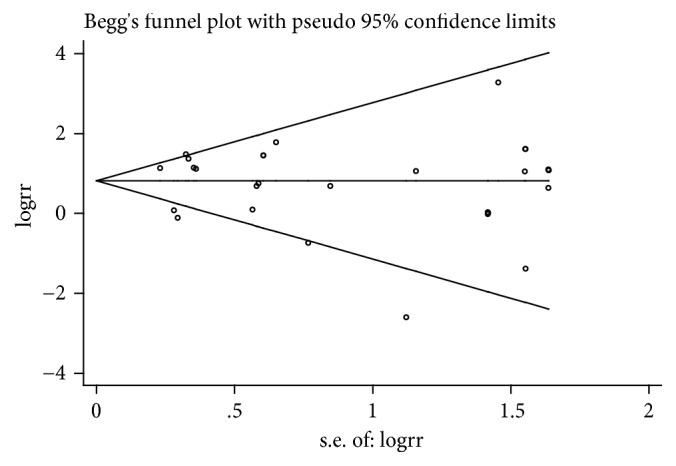
Begg's test funnel plot for the association between TLR2 Arg753Gln polymorphism and TB risk in allele model was used to detect publication bias. Each point represents an individual study for the indicated association.

Begg's test funnel plot and Egger's test were performed to assess the publication bias. The shape of the funnel plots (Figure 4) seemed symmetrical, which suggest there was no publication bias among all included studies. As shown in Table 3 there was no publication bias in allele genetic model (A vs. G: Begg's test, P=0.64; Egger's test, P=0.69), dominant genetic model (AA+AG vs. GG: Begg's test, P=0.44; Egger's test, P=0.05), and heterozygote genetic model (AG vs. GG: Begg's test, P=0.46; Egger's test, P=0.06).

## 4. Discussion

Genetics play an important role in determining risk for TB. There are more and more association studies searching susceptibility genes involved in TB. TLR2 is a crucial innate immunity gene in modulating host susceptibility. To date, several single nucleotide polymorphisms (SNPs) in TLR2 encoding gene associated with susceptibility to tuberculosis have been verified. TLR2 Arg753Gln gene polymorphism is one of the most characterized variants that are associated with susceptibility to tuberculosis. The influence of TLR2 Arg753Gln gene polymorphism on TB susceptibility has been extensively investigated in a lot of studies. Nevertheless, the results are inconsistent. Most studies have found a significant association between TLR2 Arg753Gln gene polymorphism and high TB risk across different ethnic groups, whereas the study from Ma-c et al. found that TLR2 Arg753Gln gene polymorphism was associated with lower risk to TB [2]. Furthermore, the study by Xue et al. found that TLR2 Arg753Gln gene polymorphism was not associated with susceptibility to TB [11]. The conclusions of these studies are limited due to relatively small sample size or the discrepancy of population.

Meta-analysis can pool data from individual association studies to increase the sample size under investigation; thus the statistical power of the analysis for the estimation of genetic effects was enhanced [28]. Up to now, there are five previous meta-analyses which have been done on the association between TLR2 Arg753Gln polymorphism and TB [29–33]. However, the number of original studies regarding this issue has sharply increased in the past three years. Therefore, it is necessary to perform an updated meta-analysis to increase the reliability of the results and minimize potential publication bias.

On the basis of 23 eligible studies providing data on the TLR2 Arg753Gln polymorphism and TB risk, we find significant association between the TLR2 Arg753Gln polymorphism and high TB risk in allele genetic model, dominant genetic model, and heterozygote genetic model. The AA, AG+AA genotype, and A allele, in the heterozygote, dominant, and allelic model increased TB susceptibility overall. For the pooled data, the results were consistent with most previous studies to a large extent [29–33]. In the subgroup analysis based on ethnicity, the subgroup results were inconsistent with the overall outcome among different ethnic populations. A significant association was found in Asian populations, but a nonsignificant association was found in African and mixed populations. This result suggests that gene polymorphisms might lead to ethnic-specific susceptibility to TB. However, due to the limitation of small sample size of the study in African and Mixed population, further investigations should be conducted in different ethnicities to clarify this issue. The heterogeneity was observed in allele model, but not in dominant or heterozygote model. Subgroup analysis by ethnicity found was conducted to identify the source of heterogeneity. After omission of the study on mixed population, the heterogeneity overall was resolved (Figure S1). These results suggested the study on mixed populations may account for the overall heterogeneity.

The stratified analyses by the HWE in the controls showed TLR2 Arg753Gln polymorphism significantly increased TB risk in either subgroup, which were consistent with the overall outcome. Deviation from HWE did not affect the conclusions of this meta-analysis. However, high heterogeneity existed in the subgroup following HWE in the controls, which could reduce the reliability of this result. Thus, it is necessary to carry out further research to verify this point. Furthermore, in the subgroup analysis by sample type TLR2 Arg753Gln polymorphism significantly increased TB risk, but not EPTB. It is worth noting that patients are diagnosed as PTB or EPTB, whereas there were a small proportion of patients diagnosed with TB which included both PB and EPTB. Their results should be interpreted with more caution.

Publication bias is an important factor affecting the quality of meta-analysis. In order to assess publication bias, Begg's test funnel plot and Egger's test were performed. There was no publication bias observed in allele genetic model, dominant genetic model, or heterozygote genetic model.

There are some advantages in our meta-analysis. First, this meta-analysis had larger sample sizes compared to any previous meta-analysis. Thus our results are more reliable and powerful. Second, more ethnic populations, such as African descent and mixed descent, were included in our study compare to previous study, so our conclusion might be more conclusive.

Nevertheless, there are still some limitations in this meta-analysis. First, all included article has been published in English or Chinese. Other language or unpublished studies might not be included in this meta-analysis, which make our analyses prone to generate potential publication bias. Second, in subgroup analysis, there were only 2 studies included in African populations and 3 studies included in mixed populations, which may obscure the subgroup results. So, more original studies from different populations such as African are needed to make our conclusions reliable and accurate. Third a lot of studies lacked detailed information such as sex, gender, etc., so the pooled results in this meta-analysis were not based on adjustment estimates. Fourth, different genotyping methods are used among various study included in this meta-analysis, such as PCR-RFLP assay, PCR-SSP, MALDI-TOFMS, ARMS-PCR, SNaPshot, DNA sequencing, and Taqman. Each method has its own limitation in the way of sensitivity, or performance stability, or instrumental error etc., which might affect the individual results.

In conclusion, this meta-analysis aimed to summarize association between the TLR2 Arg753Gln gene polymorphism and TB susceptibility. We found that TLR2 Arg753Gln gene polymorphism increases susceptibility to TB in overall, which support the fact that TLR2 plays an important role in host immune response against tuberculosis. More studies in different ethnic groups are required to be done to reinforce the results of this meta-analysis. Nevertheless, gene-gene or gene-environment interaction which is closely related to the susceptibility of TB should be considered in future studies.

## Figures and Tables

**Table 1:** Individual characteristics of the studies included in this meta-analysis.

Author	Year	Sample types	Country	Ethnicity	Genotyping method	Sample size		HWE
						(case/control)		
						case	control	
Ogus-a [6]	2004	PTB	Turkey	Caucasian	PCR-RFLP	151	116	≤0.01
Ogus-b [6]	2004	EPTB	Turkey	Caucasian	PCR-RFLP	129	116	≤0.01
Jin [7]	2006	PTB	China	Asian	PCR-SSP	170	199	0.23
Ma-a [2]	2007	TB	African Americans	African	Sequencing	339	194	NA
Ma-b [2]	2007	TB	European American	Caucasian	Sequencing	180	110	0.81
Ma-c [2]	2007	TB	Hispanics	Mixed	Sequencing	375	114	0.85
Yu [8]	2008	TB	China	Asian	PCR-RFLP	77	75	NA
Biswas [9]	2009	PTB	India	Asian	PCR-RFLP	100	100	NA
Strapagiel [10]	2008	TB	Poland	Caucasian	PCR-RFLP	79	62	0.90
Xue [11]	2010	PTB	China	Asian	Sequencing	205	203	0.97
Selvaraj [12]	2010	PTB	Indian	Asian	PCR-RFLP	193	199	0.97
Ma [13]	2010	PTB	China	Asian	Sequencing	543	544	NA
Etokebe [14]	2010	PTB	Croatia	Caucasian	Taqman	103	105	0.96
Le [15]	2011	PTB	China	Asian	PCR-SSP	225	422	0.92
Dalgic-a [16]	2011	TB	Turkey	Caucasian	PCR-RFLP	138	200	0.61
Dalgic-b [16]	2011	PTB	Turkey	Caucasian	PCR-RFLP	60	200	0.1
Dalgic-c [16]	2011	EPTB	Turkey	Caucasian	PCR-RFLP	138	200	0.61
Sánchez [17]	2012	PTB	Columbia	Caucasian	MALDI-TOFMS	466	300	0.91
Torres-García [18]	2013	PTB	Mexican	Caucasian	Taqman	90	90	NA
Wu-a [19]	2015	TB	China	Asian	PCR-RFLP	225	422	0.92
Wu-b [19]	2015	PTB	China	Asian	PCR-RFLP	109	422	0.92
Salie [20]	2015	PTB	South African	African	NA	729	487	0.91
Jafari [21]	2015	PTB	Iranian	Asian	ARMS-PCR	96	122	0.93
Panwar-a [22]	2016	PTB	India	Asian	ARMS-PCR	106	106	NA
Panwar-b [22]	2016	PTB	India	Asian	ARMS-PCR	106	106	NA
Rizvi-a [23]	2016	EPTB	India	Asian	ARMS-PCR	130	130	NA
Rizvi-b [23]	2016	PTB	India	Asian	ARMS-PCR	130	130	NA
Saleh-a [24]	2017	PTB	Egypt	Mixed	PCR-RFLP	52	50	0.59
Saleh-b [24]	2017	EPTB	Egypt	Mixed	PCR-RFLP	44	50	0.59
Sun [25]	2017	PTB	China	Asian	SNaPshot	214	205	0.97
Mittal [26]	2018	PTB	Northern indian	Asian	PCR-RFLP	155	98	NA
Soeroto [27]	2018	PTB	Indonesian	Asian	PCR-RFLP	86	114	NA

PTB: pulmonary tuberculosis; EPTB: extra pulmonary tuberculosis; PCR-RFLP: polymerase chain reaction-restriction fragment length polymorphism; ARMS-PCR: amplification refraction mutation system-polymerase chain reaction; PCR-SSP (sequence specific primer); MALDI-TOFMS (matrix-assisted laser desorption/ionization time-of-flight mass spectrometry); ARMS-PCR (the amplification refractory mutation system); HWE: Hardy-Weinberg equilibrium; SSCP, single strand conformation polymorphism; NA, not available.

**Table 2:** Genotype and allele distribution of TLR2 Arg753Gln gene polymorphism in TB and controls.

Author	Year	Case					Control				
		G	A	GG	GA	AA	G	A	GG	GA	AA
Ogus-a [6]	2004	261	41	124	13	14	221	11	107	7	2
Ogus-b [6]	2004	224	34	106	12	11	221	11	107	7	2
Jin [7]	2006	269	71	99	71	0	367	31	168	31	0
Ma-a [2]	2007	676	2	337	2	0	388	0	194	0	0
Ma-b [2]	2007	351	9	171	9	0	215	5	105	5	0
Ma-c [2]	2007	749	1	374	1	0	224	4	110	4	0
Yu [8]	2008	153	1	76	1	0	150	0	75	0	0
Biswas [9]	2009	200	0	100	0	0	200	0	100	0	0
Strapagiel [10]	2008	153	5	74	5	0	122	2	60	2	0
Xue [11]	2010	409	1	204	1	0	405	1	202	1	0
Selvaraj [12]	2010	385	1	192	1	0	397	1	198	1	0
Ma [13]	2010	1086	0	543	0	0	1088	0	544	0	0
Etokebe [14]	2010	205	1	102	1	0	209	1	104	1	0
Le [15]	2011	441	9	216	9	0	840	4	418	4	0
Dalgic-a [16]	2011	238	38	100	38	0	386	14	186	14	0
Dalgic-b [16]	2011	217	31	93	31	0	386	14	186	14	0
Dalgic-c [16]	2011	52	4	24	4	0	386	14	186	14	0
Sánchez [17]	2012	929	3	463	3	0	596	4	296	4	0
Torres-García [18]	2013	180	0	90	0	0	180	0	90	0	0
Wu-a [19]	2015	441	9	216	9	0	840	4	418	4	0
Wu-b [19]	2015	212	6	103	6	0	840	4	418	4	0
Salie [20]	2015	864	12	426	12	0	572	4	284	4	0
Jafari [21]	2015	192	0	96	0	0	242	2	120	2	0
Panwar-a [22]	2016	210	2	104	2	0	212	0	106	0	0
Panwar-b [22]	2016	211	1	105	1	0	212	0	106	0	0
Rizvi-a [23]	2016	258	2	128	2	0	260	0	130	0	0
Rizvi-b [23]	2016	259	1	129	1	0	260	0	130	0	0
Saleh-a [24]	2017	53	51	6	41	5	53	47	15	23	12
Saleh-b [24]	2017	49	39	6	37	1	53	47	15	23	12
Sun [25]	2017	425	3	211	3	0	409	1	204	1	0
Mittal [26]	2018	309	1	154	1	0	196	0	98	0	0
Soeroto [27]	2018	163	9	77	9	0	228	0	114	0	0

**Table 3:** Quantitative analyses results of the association between TLR2 Arg753Gln gene polymorphism and TB susceptibility in different genetic models.

Genetic model	OR(95%CI)	P	I^2^ (%)	Effect model	P (*Q-test*)	Begg's test	Egger's test
A vs. G	2.20 (1.60, 3.04)	≤0.01	47.6	R	<0.10	0.64	0.69
GA vs. GG	2.70 (2.00, 3.65)	≤0.01	26.2	R	0.01	0.46	0.06
(GA+AA)vs. GG	2.97 (2.39, 3.69)	≤0.01	19.0	F	0.18	0.44	0.05

P (*Q*-test): p value for heterogeneity; OR: odds ratio; CI: confidence interval; F: fixed-effect model; R: random-effect model.

**Table 4:** Subgroup analysis results of the association between TLR2 Arg753Gln gene polymorphism and TB risk in allele genetic models.

Variables	Subgroup	N	OR (95%CI)	P	I^2^ (%)
Ethnicity	Caucasian	9	2.62 (1.77, 3.87)	≤0.01	33.6
	Asian	15	3.35 (2.37, 4.74)	≤0.01	0.00
	African	2	2.08 (0.72, 6.03)	0.18	0.00
	Mixed	3	0.76 (0.36, 1.64)	0.49	62.9
HWE violation	Yes	19	1.89 (1.26, 2.85)	≤0.01	62.3
	No	10	3.30 (2.11, 5.17)	≤0.01	0.00
Sample type	PB	18	2.43 (1.66, 3.54)	≤0.01	34.1
	EPTB	4	1.84 (0.83, 4.06)	0.13	62.0
	TB (PB+EPTB)	7	1.84 (0.74, 4.59)	0.19	62.2

N: number; OR: odds ratio; CI: confidence interval.

## Supplementary Materials

Supplementary Materials
** Figure S1.** Forest plot of TB risk associated with TLR2 Arg753Gln polymorphism after omission the study on mixed population (A vs. G).
