# Supplementary material for: TLR2 Arg753Gln Gene Polymorphism Associated with Tuberculosis Susceptibility: An Updated Meta-Analysis
Source: Biomed Res Int. 2019 Jan 13;2019:2628101. doi: 10.1155/2019/2628101 (PMC6348792; doi:10.1155/2019/2628101)
Supplement: Supplementary Materials — Figure S1. Forest plot of TB risk associated with TLR2 Arg753Gln polymorphism after omission the study on mixed population (A vs. G). [file 2628101.f1.pdf]

FigureS1. Forest plot of TB risk associated with TLR2 Arg753Gln polymorphism after omission the study on mixed population (A *vs.* G).

FigureS1.

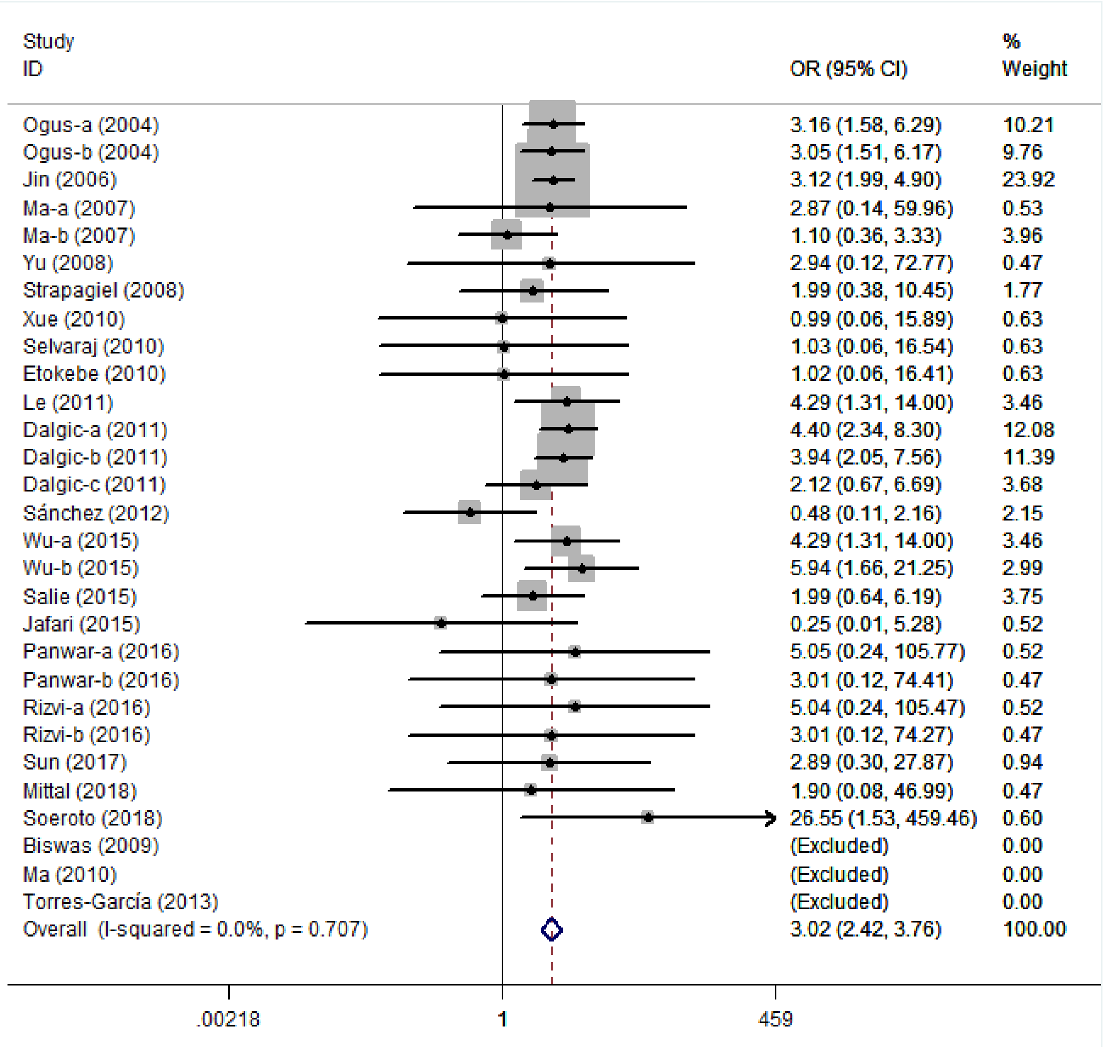
Study
ID
OR (95% CI)
%
Weight
Ogus-a (2004) 3.16 (1.58, 6.29) 10.21
Ogus-b (2004) 3.05 (1.51, 6.17) 9.76
Jin (2006) 3.12 (1.99, 4.90) 23.92
Ma-a (2007) 2.87 (0.14, 59.96) 0.53
Ma-b (2007) 1.10 (0.36, 3.33) 3.96
Yu (2008) 2.94 (0.12, 72.77) 0.47
Strapagiel (2008) 1.99 (0.38, 10.45) 1.77
Xue (2010) 0.99 (0.06, 15.89) 0.63
Selvaraj (2010) 1.03 (0.06, 16.54) 0.63
Etokebe (2010) 1.02 (0.06, 16.41) 0.63
Le (2011) 4.29 (1.31, 14.00) 3.46
Dalgic-a (2011) 4.40 (2.34, 8.30) 12.08
Dalgic-b (2011) 3.94 (2.05, 7.56) 11.39
Dalgic-c (2011) 2.12 (0.67, 6.69) 3.68
Sánchez (2012) 0.48 (0.11, 2.16) 2.15
Wu-a (2015) 4.29 (1.31, 14.00) 3.46
Wu-b (2015) 5.94 (1.66, 21.25) 2.99
Salie (2015) 1.99 (0.64, 6.19) 3.75
Jafari (2015) 0.25 (0.01, 5.28) 0.52
Panwar-a (2016) 5.05 (0.24, 105.77) 0.52
Panwar-b (2016) 3.01 (0.12, 74.41) 0.47
Rizvi-a (2016) 5.04 (0.24, 105.47) 0.52
Rizvi-b (2016) 3.01 (0.12, 74.27) 0.47
Sun (2017) 2.89 (0.30, 27.87) 0.94
Mittal (2018) 1.90 (0.08, 46.99) 0.47
Soeroto (2018) 26.55 (1.53, 459.46) 0.60
Biswas (2009) (Excluded) 0.00
Ma (2010) (Excluded) 0.00
Torres-García (2013) (Excluded) 0.00
Overall (I-squared = 0.0%, p = 0.707) 3.02 (2.42, 3.76) 100.00
.00218
1
459
